# Risk of Alzheimer's disease is associated with longitudinal changes in plasma biomarkers in the multi‐ethnic Washington Heights–Hamilton Heights–Inwood Columbia Aging Project (WHICAP) cohort

**DOI:** 10.1002/alz.13652

**Published:** 2024-01-06

**Authors:** Yian Gu, Lawrence S. Honig, Min Suk Kang, Aanya Bahl, Danurys Sanchez, Dolly Reyes‐Dumeyer, Jennifer J. Manly, Jeffrey L. Dage, Rafael A. Lantigua, Adam M. Brickman, Badri N. Vardarajan, Richard Mayeux

**Affiliations:** ^1^ Taub Institute for Research on Alzheimer's Disease and the Aging Brain, Vagelos College of Physicians and Surgeons, Columbia University New York New York USA; ^2^ G.H. Sergievsky Center, Vagelos College of Physicians and Surgeons, Columbia University New York New York USA; ^3^ Department of Neurology Vagelos College of Physicians and Surgeons Columbia University and the New York Presbyterian Hospital New York New York USA; ^4^ Department of Epidemiology Mailman School of Public Health Columbia University New York New York USA; ^5^ Stark Neurosciences Research Institute, Indiana University School of Medicine Indianapolis Indiana USA; ^6^ Department of Medicine Vagelos College of Physicians and Surgeons, Columbia University and the New York Presbyterian Hospital New York New York USA

**Keywords:** Alzheimer's disease, amyloid, blood biomarkers, cognition, community, dementia, glial fibrillary acidic protein, longitudinal study, muti‐ethnic, neurofilament light chain, tau

## Abstract

**BACKGROUND:**

Alzheimer's disease (AD) biomarkers can help differentiate cognitively unimpaired (CU) individuals from mild cognitive impairment (MCI) and dementia. The role of AD biomarkers in predicting cognitive impairment and AD needs examination.

**METHODS:**

In 628 CU individuals from a multi‐ethnic cohort, amyloid beta (Aβ)42, Aβ40, phosphorylated tau‐181 (p‐tau181), glial fibrillary acidic protein (GFAP), and neurofilament light chain (NfL) were measured in plasma.

**RESULTS:**

Higher baseline levels of p‐tau181/Aβ42 ratio were associated with an increased risk of incident dementia. A biomarker pattern (with elevated Aβ42/Aβ40 but low p‐tau181/Aβ42) was associated with decreased dementia risk. Compared to CU, participants who developed MCI or dementia had a rapid decrease in this protective biomarker pattern reflecting AD‐specific pathological change.

**DISCUSSION:**

Elevated levels of AD biomarker p‐tau181/Aβ42, by itself or combined with a low Aβ42/Aβ40 level, predicts clinically diagnosed AD. Individuals with a rapid change in these biomarkers may need close monitoring for the potential downward trajectory of cognition.

**Highlights:**

- We discuss a multi‐ethnic, urban community study of elderly individuals.
- The study consisted of a longitudinal assessment over 6 years with repeated clinical assessments.
- The study used blood‐based biomarkers as predictors of mild cognitive impairment and Alzheimer's disease.

## 1 BACKGROUND

Blood‐based biomarkers for Alzheimer's disease (AD), including amyloid beta (Aβ), tau, neurofilament light chain (NfL), and glial fibrillary acidic protein (GFAP), circulating molecular signatures of the amyloid, tau, and neurodegeneration (ATN) and inflammation, support their use in research and in clinical settings.^1^ Compared to cerebrospinal fluid (CSF) and positron emission tomography (PET) biomarkers,^2^, ^3^ blood‐based biomarkers are less invasive, easily accessible, less expensive, and more suitable for large epidemiological studies and clinical trials. Potential clinical applications of these blood‐based biomarkers include application in diagnosis, disease monitoring and prognosis, treatment management, screening, early detection, as well as risk prediction.^4^

To date, much of the research on blood‐based biomarkers has focused on their diagnostic value in research and in specialized settings.^5^, ^6^, ^7^, ^8^, ^9^, ^10^, ^11^, ^12^ Data from highly selected participants have been used for developing optimal thresholds for cut points, based on the presence or absence of AD pathology in autopsy or amyloidosis in PET imaging as gold standards.^13^, ^14^, ^15^, ^16^ However, to date, no universally standardized and validated diagnostic cut points have been established, nor have blood‐based biomarkers been widely used to monitor the disease progression or evaluate treatment responses. In addition to these applications, blood‐based biomarkers could also aid in the identification of cognitively unimpaired (CU) individuals at risk of developing dementia. Blood‐based biomarkers may also be of value as antecedent risk factors in prediction of mild cognitive impairment (MCI) and AD and related dementias (ADRD) in asymptomatic individuals.

ADRD is known to have a long preclinical phase. Many of the neuropathological brain imaging changes occur during this preclinical stage. Thus, longitudinal changes in biomarker levels in large, population‐based, ethnically diverse cohorts would augment the value of blood‐based biomarkers. Here, we examined whether blood‐based biomarkers measured before the onset of clinical symptoms can predict the development of clinically diagnosed MCI or AD. This approach would help identify individuals at risk for disease‐modifying treatments, augment studies examining biological mechanisms by identifying critical biomarker targets, and help identify modifiable factors that work through these biomarkers to delay the onset of the disease.

Using data from the Washington Heights–Hamilton Heights–Inwood Columbia Aging Project (WHICAP) study, a longitudinal community‐based, multi‐ethnic population of older adults, we examined whether the initial measurement of blood‐based biomarkers could predict subsequent MCI or AD diagnosis. We also investigated whether the rate of change in blood‐based biomarkers over time differed among cognitively unimpaired individuals and those with newly diagnosed MCI or AD.

## 2 METHODS

### 2.1 Study population

WHICAP is a multi‐ethnic, community‐based, prospective cohort study of clinical and genetic risk factors for dementia. Three waves of individuals were recruited in 1992, 1999, and 2009 in WHICAP, all using similar study procedures.^17^, ^18^ Briefly, participants were recruited as representatives of individuals living in the communities of northern Manhattan and were ≥ 65 years. At the study entry, each person underwent a structured interview of general health and function, followed by a comprehensive assessment including medical, neurological, and psychiatric histories, and standardized physical, neurological, and neuropsychological examinations. Individuals were followed every 18 to 24 months, repeating examinations that were similar to baseline.

The institutional review boards of Columbia University gave ethical approval for this work. All participants provided written informed consent.

For this specific analysis, we selected individuals when they met the following criteria: (1) indicated that they had not been diagnosed with AD or a related disorder at the initial interview; (2) had at least three blood samples at three different study follow‐up visits; and (3) after each WHICAP follow‐up visit had a clinical diagnosis of being CU, MCI,^19^ or dementia.^20^ All individuals had three clinical visits that included blood sampling for biomarkers. For individuals whose diagnosis status changed over the WHICAP clinical follow‐up visits, the baseline visit, the incident MCI or dementia visit, and a third visit were selected. For participants remaining CU through the follow‐up, the baseline, the most recent, and a middle visit were chosen.

### 2.2 Cognitive assessment and clinical diagnosis of AD

At each WHICAP visit, individuals underwent a standardized neuropsychological battery^21^ administered either in English or Spanish at baseline and each follow‐up visit. Composite *z* scores for four cognitive domains (memory, language/executive, speed, and visuospatial) were calculated based on a factor analysis using principal axis factoring and oblique rotation^21^ on neuropsychological test scores. The resulting factor structure and factor loadings were invariant across English and Spanish speakers.^22^

All diagnoses were made in a diagnostic consensus conference attended by a panel consisting of at least one neurologist and one neuropsychologist with expertise in dementia diagnosis, using results from the neuropsychological battery and evidence of impairment in social or occupational function. All‐cause dementia was determined based on Diagnostic and Statistical Manual of Mental Disorders, 4th Edition criteria.^20^ Furthermore, we used the criteria from the National Institute of Neurological and Communicative Disorders and Stroke—Alzheimer's Disease and Related Disorders Association to diagnose probable or possible AD.^23^ Incident dementia was identified when the participants were clinically diagnosed with dementia for the first time during follow‐up among those who did not have dementia at baseline. For participants without dementia, MCI was assigned, as previously described,^19^ if the participant had memory complaints, had cognitive impairment in one or more cognitive domains, but with preserved activities of daily living. For all analyses, we combined MCI with dementia patients first and then examined the incident MCI and dementia separately compared to CU.

### 2.3 Plasma biomarkers

Blood samples were collected by standard venipuncture in dipotassium ethylenediaminetetraacetic acid tubes. Plasma was prepared by centrifugation at 2000 × g for 15 minutes at 4°C within 2 hours after collection, aliquoted in polypropylene tubes, and frozen and stored at −80°C. Blood for DNA extraction was also collected, and apolipoprotein E (*APOE*) genotyping was performed at LGC Genomics and CD Genomics.

**RESEARCH IN CONTEXT**

1. **Systematic review**: Few studies have evaluated the clinical application of Alzheimer's disease (AD) blood‐based biomarkers longitudinally as antecedent risk predictors. Data from multi‐ethnic populations are even more limited. How preclinical trajectories of blood‐based biomarkers are related to the risk of developing clinically diagnosed mild cognitive impairment (MCI) or AD is largely unknown.
2. **Interpretation**: High circulating levels of phosphorylated tau 191 (p‐tau181)/amyloid beta (Aβ)42, by itself or combined with a low level of Aβ42/Aβ40, may predict development of incident clinical AD. Biomarkers levels of p‐tau181, p‐tau181/Aβ42, and neurofilament light chain increase with age even among individuals who remain cognitively healthy. A rapid change in biomarkers may indicate the individuals in the active trajectory to develop clinically diagnosed MCI or AD.
3. **Future directions**: Larger studies or meta‐analyses are needed to examine whether the predictive utility of blood‐based biomarkers for AD differs across racial/ethnic groups. Well‐designed studies are needed to evaluate the optimal duration between repeated measures of biomarkers.

Plasma biomarker assays were performed between April 2022 and November 2022 using the single molecule array technology Quanterix Simoa (single molecule array)^24^ HD‐X platform (Quanterix). Samples were diluted and assayed in duplicate per package insert instructions using three Quanterix kits: Neurology 3‐Plex A (catalog No. 101995) for Aβ42, Aβ40, and total tau (t‐tau); P‐tau181 V2 Advantage (catalog No. 103714) for tau phosphorylated at threonine 181 (p‐tau181); and Neurology 2‐Plex B (catalog No. 103520) for NfL and GFAP. Quantification functional lower limits for these analytes are 2.7 for Aβ40, 0.6 for Aβ42, 0.3 for t‐tau, 0.3 for p‐tau181, 0.8 for NfL, and 16.6 for GFAP, all in pg/mL. More than 5000 assays were conducted for these analytes, and mean coefficients of variation (CV%) are ≤ 5%. Ratios of Aβ42/Aβ40 and p‐tau181/Aβ42 were calculated. Based on the literature, we a priori decided to focus on p‐tau181,^25^ neurodegeneration marker NfL,^10^, ^26^, ^27^ neuroinflammatory reactive astrogliosis marker GFAP,^28^ Aβ42/Aβ40,^29^, ^30^, ^31^ and P‐tau181/Aβ42,^32^ while Aβ42, Aβ40, and t‐tau were not investigated due to their limited value.^33^, ^34^

### 2.4 Covariates

Demographic data including age (years), sex (women, men), and ethnic group (White non‐Hispanic, Black, Hispanic, and others), and education (years), were collected at the initial interview. *APOE* ε4 genotype was defined based on the presence (either one or two) of ε4 alleles. We examined the most prevalent individual traits and comorbidities, including hypertension, diabetes, heart disease, depression, and arthritis, based on self‐reported medical history and/or current medication use. Subjects who had ever smoked one or more cigarettes per day for a period of ≥ 1 year were regarded as smokers.^35^ Body mass index (BMI) was calculated as weight in kilograms divided by height in meters squared, with weight and height measured at the clinical visits, and was subsequently categorized into underweight or normal (<25 kg/m^2^), overweight (≥25 kg/m^2^ and <30 kg/m^2^), or obese (≥30 kg/m^2^).

In a subset sample of the study, we measured plasma creatinine using a kinetic colorimetric assay on an automated analyzer (Roche Integra 400 plus) at the Clinical Research Resource lab in the Irving Institute for Clinical and Translational Research, Columbia University Irving Medical Center. A creatinine level ≥ 1.3 mg/dL for men or ≥ 1.0 mg/dL for women was considered an indication of renal dysfunction.^36^

### 2.5 Statistical analysis

Descriptive statistics for individual demographic and clinical characteristics and plasma biomarker levels were compared among CU, incident MCI, and incident AD participants using χ^2^ for categorical variables and Kruskal–Wallis tests or analysis of variance (ANOVA) for continuous variables. Because the distributions of biomarkers were skewed, log‐transformed biomarker levels were used in the analyses. For better visualize the biomarker levels, *Z* scores of the log‐transformed biomarkers were used so the effect sizes could be compared among the biomarkers. Pearson's correlations among the biomarkers as well as age, education, and BMI were examined. Biomarker levels were also compared between men and women, *APOE* ε4 carriers and non‐carriers, and across ethnicity groups using ANOVA.

We used Cox proportional hazard models to examine whether baseline biomarker level could predict clinically diagnosed MCI and AD. The time variable was defined as the duration from the baseline to the last follow‐up blood collection dates for controls, and the duration from the baseline to the incident MCI/AD diagnosis for those who developed MCI/AD. Analyses were adjusted for age, education, sex, and ethnic group (model 1). In model 2, *APOE* ɛ4 status, and Charlson Comorbidity Index (CCI) were additionally adjusted. The individual biomarkers (p‐tau181, NfL, GFAP, Aβ42/Aβ40, and pP‐tau181/Aβ42 ) were included in Cox models separately. Similar analyses were performed to examine the risk of incident MCI (incident AD was censored) and incident AD separately.

We used generalized estimating equations (GEE) models with repeated biomarker measures as outcomes to examine whether levels changed over time and whether individuals with incident MCI/AD and CU had different rates of change in plasma biomarkers. We used the duration from the baseline to the follow‐up blood collection dates as the time variable. Models were adjusted with the same covariates as in the Cox models. Similar analyses were performed to examine the difference between CU and incident MCI, and between CU and incident AD separately. Similar GEE models were also used to explore factors that are associated with rates of biomarker change over time among CU participants.

We performed supplementary analyses to assess the combined effects of biomarkers^6^ as a predictor of disease status. We performed principal component analysis (PCA) on the correlation matrix of NfL, GFAP, Aβ42/Aβ40, and p‐tau181/Aβ42. The number of patterns to be retained was determined by eigenvalues > 1.0, scree plot, parallel analysis, and interpretability of the factors. We performed the PCA at each visit separately. We considered biomarkers with an absolute factor loading value ≥ 0.30 on a pattern as dominant biomarkers contributing to that biomarker pattern. The patterns derived from the three visit‐specific PCAs were similar, each having the first two patterns (PCA1 and PCA2) retained, which explained a total of 66%, 71%, and 71% variations of all the four biomarkers for visit 1, 2, and 3, respectively. For all visits, NfL (loadings 0.85), GFAP (loadings 0.81 to 0.87), and p‐tau181/Aβ42 (loadings 0.36–0.48) had positive loadings for the first pattern (PCA1), while Aβ42/Aβ40 had a positive loading (loadings around 0.9) and p‐tau181/Aβ42 had a negative loading (−0.3 to −0.7) for PCA2 (Table S1 in supporting information). Each person received a pattern score (i.e., a linear combination of biomarker weighted by factor loadings) for each identified biomarker pattern. Thus, a higher PCA1 score would indicate a higher likelihood of neuronal injury, neuroinflammatory and neurodegenerative profile,^37^, ^38^ and a higher PCA2 score, in contrast, would indicate a lower likelihood of AD‐specific pathological changes. We used the PCA1 and PCA2 scores in the above Cox and GEE models to examine their predicting roles for AD and/or MCI.

Sensitivity analyses were performed to limit the GEE analysis to the pre‐diagnosis visits only. We excluded samples with CV% larger than 15% in sensitivity analyses. Instead of using the CCI, we simultaneously included the prevalent individual diseases or conditions (hypertension, diabetes, heart disease, arthritis, depression, and smoking) in the adjusted Cox or GEE models. We performed interaction analysis to examine whether the associations differed by ethnicity, sex, and *APOE* ɛ4 status.

Two‐sided statistical tests were conducted, and *P* < 0.01 with Bonferroni correction for multiple comparison (5 biomarkers: p‐tau181, NfL, GFAP, Aβ42/Aβ40, and p‐tau181/Aβ42) was considered statistically significant. Statistical analyses were conducted with SPSS.

## 3 RESULTS

### 3.1 Descriptive analysis

The current study included the first 628 CU individuals selected from eligible WHICAP participants who met the above criteria. At visit 2, which was on average 3.6 years from the baseline, 126 (20% of 628) converted from CU to MCI by clinical diagnosis, 16 (2.5%) converted to AD by clinical diagnosis, and 486 (77%) remained to be CU; at visit 3, which was 6.96 years from the baseline, an additional 72 (15% of 486) CU had converted to MCI, 8 (1.6% of 486) converted to AD from CU, and 33 (26% of 126) MCI further converted to AD. Overall, 165 (26% of 628) individuals developed MCI, 57 (9%) developed AD, and 406 (65%) remained CU during an average 6.96 (standard deviation [SD] = 3.07) years of follow‐up. A total number of 585 (380, 151, 53 of CU, MCI, AD, respectively) had blood draws from all three visits, but 43 (7%; 26, 13, 4 of CU, MCI, AD, respectively) had two samples only as one of their samples was degraded and could not be used to measure biomarker concentrations reliably.

The mean age of individuals at the initial visit was 73.4 (SD = 5.6) years, 427 (67.9%) were women, and 20.4% carried one or two *APOE* ɛ4 alleles. Individuals self‐identified as non‐Hispanic White (27.7%), Black (25%), Hispanic (45.2%), or non‐Hispanic people who did not identify as White or Black (2.1%).

Compared to CU individuals, those who developed either MCI or AD were older; were more likely to be Hispanic; had more comorbidities (specifically, hypertension and arthritis); and had higher levels of p‐tau181, NfL, GFAP, and higher p‐tau181/Aβ42. There was no difference in the level of Aβ42/Aβ40 (Table 1).

**TABLE 1:** Characteristics of the study participants according to the disease outcome during follow‐up.

	Cognitively unimpaired	Incident MCI	Incident AD	Total	
	(*N* = 406)	(*N* = 165)	(*N* = 57)	(*N* = 628)	*P* ^*^
Age (years), mean (SD)	71.8 (5.00)	75.7 (5.04)	78.2 (5.92)	73.4 (5.58)	<0.0001
Duration of storage time, mean (SD)	15.54 (7.7)	20.70 (9.58)	21.97 (9.22)	17.48 (8.75)	<0.0001
Duration between visit 1 and 2, mean (SD)	3.16 (1.64)	4.40 (2.86)	4.26 (2.29)	3.58 (2.16)	<0.0001
Duration between visit 1 and 3, mean (SD)	6.35 (2.77)	8.03 (3.41)	8.21 (2.93)	6.96 (3.07)	<0.0001
Duration of follow‐up time for disease outcome, mean (SD)	6.16 (2.73)	8.03 (4.45)	6.91 (2.87)	6.72 (3.38)	<0.0001
Female, N (%)	275 (67.7)	112 (67.9)	40 (70.2)	427 (68.0)	0.933
Race/ethnicity, N (%)					<0.0001
Non‐Hispanic White	153 (37.7%)	17 (10.3%)	4 (7.0%)	174 (27.7%)	
Non‐Hispanic Black	112 (27.6%)	39 (23.6%)	6 (10.5%)	157 (25.0%)	
Hispanic	129 (31.8%)	108 (65.5%)	47 (82.5%)	284 (45.2%)	
Others	12 (3%)	1 (0.6%)	0 (0%)	13 (2.1%)	
*APOE* ɛ4 carrier, N (%)	72 (17.7%)	28 (17.0%)	16 (28.1%)	116 (18.5%)	0.036
Baseline biomarker levels:					
P‐tau181 (pg/mL), median [IQR]	2.14 [1.65‐2.90]	2.16 [1.62‐3.03]	2.78 [1.86‐3.71]	2.22 [1.69‐2.98]	0.003
Geometric mean (SD)	2.187 (1.667)	2.088 (2.090)	2.770 (1.631)	2.208 (1.790)	0.005
NfL (pg/mL), median [IQR]	17.5 [13.1‐24.7]	19.8 [14.8‐26.8]	24.3 [16.7‐30.6	18.2 [13.7‐25.9]	<0.0001
Geometric mean (SD)	18.104 (1.625)	20.203 (1.590)	23.466 (1.620)	19.077 (1.626)	<0.0001
GFAP (pg/mL), median [IQR]	138 [102‐190]	158 [112‐215]	193 [126‐261]	147 [106‐206]	0.001
Geometric mean (SD)	140.990 (1.666)	155.200 (1.619)	175.181 (1.693)	147.470 (1.663)	0.004
Aβ42/Aβ340, median [IQR]	0.044 [0.038‐0.051]	0.045 [0.041‐0.052]	0.044 [0.038‐0.047]	0.044 [0.039‐0.0519]	0.077
Geometric mean (SD)	0.044 (1.362)	0.048 (1.577)	0.044 (1.306)	0.045 (1.421)	0.014
P‐tau181/Aβ42, median [IQR]	0.332 (0.536)	0.365 (0.666)	0.855 (3.15)	0.387 (1.10)	<0.0001
Geometric mean (SD)	0.235 (1.966)	0.244 (2.099)	0.384 (2.312)	0.248 (2.057)	<0.001
Serum creatinine (mg/dl), mean (SD)	0.995 (0.347)	1.21 (0.844)	1.07 (0.341)	1.05 (0.505)	0.058
Charlson Comorbidity Index, mean (SD)	1.97 (1.45)	2.34 (1.50)	2.75 (1.53)	2.13 (1.49)	<0.0001
Hypertension, N (%)	323 (79.6%)	144 (87.3%)	53 (93%)	520 (82.8%)	0.009
Diabetes, N (%)	121 (29.8%)	57 (34.5%)	24 (42.1%)	202 (32.2%)	0.132
Heart disease, N (%)	167 (41.1%)	73 (44.2%)	24 (42.1%)	264 (42.0%)	0.792
Depression, N (%)	80 (19.7%)	47 (28.5%)	13 (22.8%)	140 22.3%)	0.073
Head injury, N (%)	77 (19.0%)	25 (15.2%)	11 (19.3%)	113 (18%)	0.541
Arthritis, N (%)	166 (40.9%)	87 (52.7%)	38 (66.7%)	291 (46.3%)	<0.0001
Ever smoked, N (%)	190 (46.8%)	67 (40.6%)	24 (42.1%)	281 (44.7%)	0.369
BMI, N (%)					0.16
Underweight or normal	81 (20.0%)	22 (13.3%)	7 (12.3%)	110 (17.5%)	
Overweight	141 (34.7%)	51 (30.9%)	20 (35.1%)	212 (33.8%)	
Obese	73 (18.0%)	40 (24.2%)	12 (21.1%)	125 (19.9%)	

Abbreviations: Aβ, amyloid beta; AD, Alzheimer's disease; *APOE*, apolipoprotein E; BMI, body mass index; GFAP, glial fibrillary acidic protein; IQR, interquartile range; MCI, mild cognitive impairment; NfL, neurofilament light chain; p‐tau181, phosphorylated tau‐181; SD, standard deviation.

^*^ *P* values were from chi‐square tests for categorical variables and analysis of variance or Kruskal–Wallis test for continuous variables.

At baseline, there were strong positive correlations among p‐tau181, NfL, GFAP, and p‐tau181/Aβ42, but they were not correlated with Aβ42/Aβ40 except for the negative correlation between p‐tau181 and Aβ42/Aβ40 (Table 2). At baseline, those who were older and those who had more comorbidities had higher biomarkers levels, with the exception of Aβ42/Aβ40 (Table 2). Women had higher levels of NfL (*P* = 0.007) and GFAP (*P* < 0.001) than men. *APOE* ɛ4 carriers had higher level of p‐tau181/Aβ42 (*P* = 0.024) than non‐carriers. Hispanics had a lower level of p‐tau181 (*P* = 0.008) than White non‐Hispanics, and Black individuals had lower level of NfL than White non‐Hispanics (*P* = 0.046).

**TABLE 2:** Correlations among biomarkers and demographic factors.

	P‐tau181	NfL	GFAP	Aβ42/Aβ40	p‐tau181/Aβ42	PCA1	PCA2	Age
P‐tau181	1.00	0.34	0.21	−0.18	0.60	0.47	−0.31	0.18
NfL	0.34^*^	1.00	0.50	0.00	0.24	0.85	0.02	0.39
GFAP	0.21^*^	0.50^*^	1.00	0.00	0.13	0.81	0.08	0.33
Aβ42/Aβ40	−0.18^*^	0.00	0.00	1.00	−0.04	0.03	0.96	0.01
p‐tau181/ Aβ42	0.60^*^	0.24^*^	0.13^*^	−0.04	1.00	0.48	−0.30	0.20
PCA1	0.47^*^	0.85^*^	0.81^*^	0.03	0.48^*^	1.00	0.00	0.44
PCA2	−0.31^*^	0.02	0.08	0.96^*^	−0.30^*^	0.00	1.00	0.00
Age	0.18^*^	0.39^*^	0.33^*^	0.01	0.20^*^	0.44^*^	0.00	1.00
BMI	0.01	−0.06	0.00	−0.01	0.11	0.00	−0.04	0.11

*Notes*: PCA1 and PCA2 were derived from principal component analysis (PCA) using log‐transformed values of Aβ42/Aβ40, p‐tau181/ Aβ42, NfL, and GFAP, with PCA1 having positive loadings on p‐tau181/ Aβ42, NfL, GFAP, and PCA2 having positive loading on Aβ42/Aβ40 and negative loading on p‐tau181/ Aβ42.

Abbreviations: Aβ, amyloid beta; BMI, body mass index; GFAP, glial fibrillary acidic protein; NfL, neurofilament light chain; p‐tau181, phosphorylated tau‐181;

^*^ *P* < 0.01. The log‐transformed values of biomarkers were used in the Pearson correlation analyses. Values in the table show the Pearson correlation coefficients.

**FIGURE 1:**
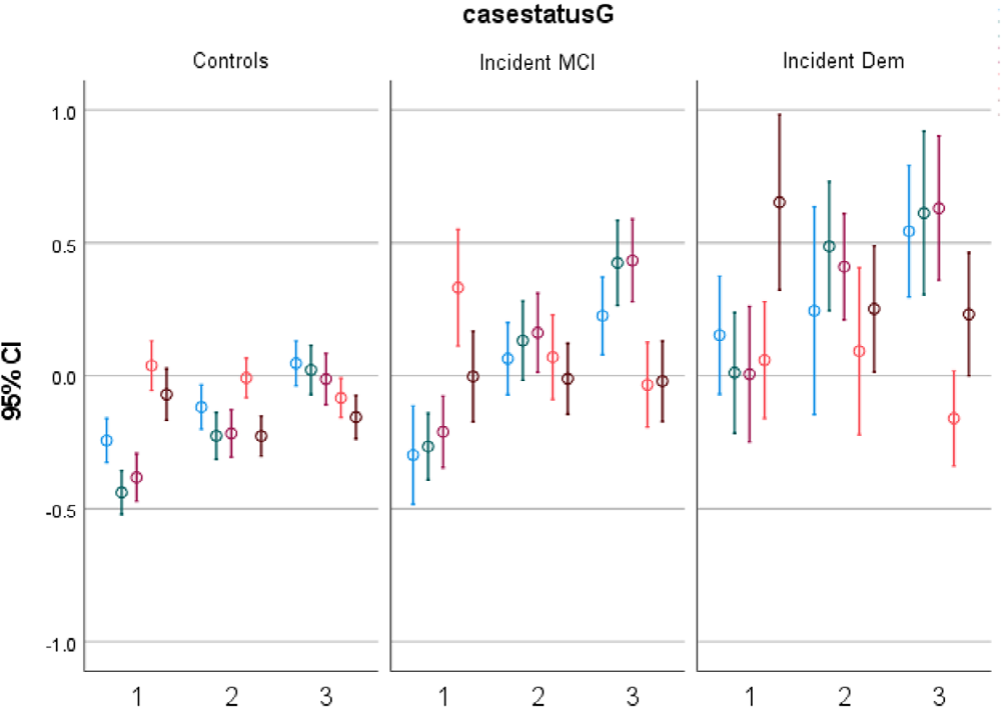
*Z* scores of the log‐transformed blood‐based biomarkers of Alzheimer's disease at different visits, among cognitively unimpaired, incident mild cognitive impairment (MCI), and incident Alzheimer's disease (Dem). Mean (95% confidence interval [CI]) of the biomarker levels (Y axis) at visits 1, 2, and 3 (X axis), in cognitively unimpaired, incident MCI, and incident dementia participants. *Z* scores of the log‐transformed biomarker levels are presented for the convenience of presentation. Blue represents Log_10_ p‐tau181, green represents Log_10_ NfL, red represents Log_10_ GFAP, orange represents Log_10_ Aβ42/Aβ40, and brown represents Log_10_ p‐tau181/Aβ42. Aβ, amyloid beta; GFAP, glial fibrillary acidic protein; NfL, neurofilament light chain; p‐tau, phosphorylated tau.

**FIGURE 2:**
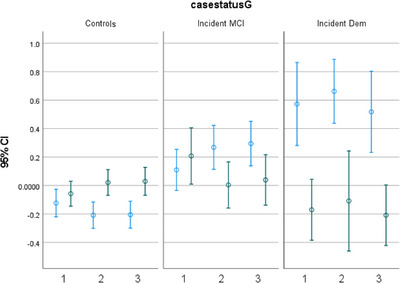
Two patterns (PCA1 and PCA2) derived from principal component analyses of the blood‐based biomarkers at the three clinical visits over the period of follow‐up. Mean (95% confidence interval [CI]) of the biomarker pattern scores (Y axis) at visits 1, 2, and 3 (X axis), in cognitively unimpaired, incident mild cognitive impairment (MCI), and incident dementia (Dem) participants. Blue represents PCA1, and green represents PCA2.

Figure 1 shows the log transformed *z* scores for each biomarker, and Figure 2 illustrates the two patterns (PCA1 and PCA2) derived from the PCAs over time at each visit. Examining the repeated measures of biomarkers among CU participants, levels of p‐tau181 (b = 0.010, *P* < 0.001), NfL (b = 0.018, *P* < 0.001), and GFAP (b = 0.015, *P* < 0.001) increased during follow‐up, adjusting for baseline age. Adjusting for baseline age, women had slower increase in p‐tau181 (β for interaction female x time = −0.012, *P* = 0.002), and Black and Hispanic individuals had faster increase than White non‐Hispanics in NfL (β for Hispanic x time = 0.010, *P* = 0.006; β for Black x time = 0.015, *P* = 0.002) and PCA1 (β for Hispanic x time = 0.043, *P* = 0.004; β for Black x time = 0.048, *P* = 0.010), and *APOE* ɛ4 carriers had faster increase in p‐tau181 ((β for interaction *APOE* x time = 0.011, *P* = 0.032).

### 3.2 Longitudinal association of the baseline blood‐based biomarkers with clinically diagnosed incident MCI/AD

In Cox models adjusted for age, sex, ethnic group, and education (Table 3, Model 1), we found higher baseline levels of p‐tau181 (hazard ratio [HR] = 4.77, 95% confidence interval [CI] = 1.52–14.95, *P* = 0.007) and p‐tau181/Aβ42 ratio (HR = 2.94, 95% CI = 1.50–5.78, *P* = 0.002) were associated with increased risk of developing incident AD by the clinical diagnosis. Additionally adjusting for *APOE* ɛ4 status and CCI, the significant association remained for p‐tau181/Aβ42 (HR = 3.13, 95% CI = 1.43–6.87, *P* = 0.004) but the association for p‐tau181 was attenuated (HR = 2.88, 95% CI = 0.79–10.56, *P* = 0.11; Table 3, Model 2). While other biomarkers did not reach significance, their associations with AD risk were all in the expected direction (Table 3, Model 2). In the supplementary analyses, the PCA1 (HR = 1.50, 95% CI = 1.12–2.01, *P* = 0.006, Table 2, Model 1) and PCA2 (HR = 0.66, 95% CI = 0.49–0.88, *P* = 0.005, Table 3, Model 1) were both associated with incident AD in Model 1, and similar results were found in Model 2.

**TABLE 3:** Association between biomarkers and incident mild cognitive impairment (MCI) or Alzheimer's disease (AD) from Cox proportional hazards models.

	Model 1^a^		Model 2^b^	
*Inc. MCI+AD*	HR (95% CI)	*P*	HR (95% CI)	*P*
P‐tau181	1.06 (0.93–1.21)	0.37	1.51 (0.88–2.59)	0.135
NfL	1.12 (0.94–1.33)	0.194	1.29 (0.57–2.92)	0.548
GFAP	1.10 (0.95–1.28)	0.211	1.72 (0.82–3.61)	0.151
Aβ42/Aβ40	0.92 (0.81–1.05)	0.206	0.24 (0.09‐0.62)	0.003
P‐tau181/Aβ42	0.93 (0.81–1.06)	0.289	0.75 (0.47—1.19)	0.217
PCA1	1.06 (0.92–1.23)	0.426	1.04 (0.88–1.23)	0.630
PCA2	0.86 (0.76–0.99)	0.029	0.84 (0.73–0.97)	0.021

***Inc. MCI***	HR (95% CI)	*P*	HR (95% CI)	*P*
P‐tau181	1.06 (0.93–1.21)	0.37	1.22 (0.67–2.23)	0.508
NfL	1.12 (0.94–1.33)	0.194	1.73 (0.73–4.13)	0.215
GFAP	1.10 (0.95–1.28)	0.211	1.69 (0.79–3.63)	0.178
Aβ42/Aβ40	0.92 (0.81–1.05)	0.206	0.67 (0.24–1.85)	0.440
P‐tau181/Aβ42	0.93 (0.81–1.06)	0.289	0.67 (0.39–1.16)	0.156
PCA1	1.07 (0.91–1.27)	0.417	1.08 (0.90–1.30)	0.401
PCA2	0.95 (0.82–1.09)	0.453	0.99 (0.85–1.15)	0.885

***Inc. AD***	HR (95% CI)	*P*	HR (95% CI)	*P*
P‐tau181	4.77 (1.52–14.95)	0.007	2.88 (0.79–10.56)	0.110
NfL	3.42 (0.81–14.48)	0.095	3.04 (0.58–15.89)	0.189
GFAP	3.49 (0.92–13.26)	0.067	3.44 (0.79–14.96)	0.099
Aβ42/Aβ40	0.10 (0.01–0.81)	0.031	0.07 (0.01–0.79)	0.031
P‐tau181/Aβ42	2.94 (1.50–5.78)	0.002	3.13 (1.43–6.87)	0.004
PCA1	1.50 (1.12–2.01)	0.006	1.49 (1.07–2.07)	0.019
PCA2	0.66 (0.49–0.88)	0.005	0.63 (0.45–0.88)	0.006

*Notes*: PCA1 and PCA2 were derived from principal component analysis (PCA) using log‐transformed values of Aβ42/Aβ40, p‐tau181/ Aβ42, NfL, and GFAP, with PCA1 having positive loadings on p‐tau181/ Aβ42, NfL, GFAP, and PCA2 having positive loading on Aβ42/Aβ40 and negative loading on p‐tau181/ Aβ42.

Abbreviations: Aβ, amyloid beta; *APOE*, apolipoprotein E; BMI, body mass index; CI, confidence interval; GFAP, glial fibrillary acidic protein; NfL, neurofilament light chain; p‐tau181, phosphorylated tau‐181.

^a^ Model 1: adjusted for age, sex, race/ethnicity, education.

^b^ Model 2: adjusted for age, sex, race/ethnicity, education, *APOE ε*4, Charlson Comorbidity Index.

### 3.3 Longitudinal analyses to examine whether the rate of blood‐based biomarkers change over time differs in cognitively healthy older adults and incident MCI/AD patients

We found a relatively faster increase of p‐tau181, NfL, GFAP, and p‐tau181/Aβ42 and faster decrease of Aβ42/Aβ40 in incident MCI/AD compared to CU participants; however, the results were not significant (Table 4). Nevertheless, incident MCI/AD participants had a different rate of change in PCA2 compared to CU participants (β = −0.036 [−0.062 to −0.011], *P* = 0.005), adjusted for age, sex, ethnic group, education, *APOE* status, and comorbidity score (Table 4, Model 2). Furthermore, similar results were found comparing incident MCI (β = −0.039 [−0.067 to −0.012], *P* = 0.005) to CU.

**TABLE 4:** Longitudinal change of biomarkers in relation to incident mild cognitive impairment (MCI) or Alzheimer's disease (AD).

	Model 1^a^				Model 2^b^			
*Inc. MCI or AD vs. CU*	B^c^	95% CI Lower Upper		*P* ^c^	B^c^	95% CI Lower Upper		*P* ^c^
P‐tau181	0.004	−0.002	0.010	*0.202*	0.005	−0.002	0.011	0.156
NfL	0.002	−0.003	0.007	*0.368*	0.002	−0.004	0.007	0.575
GFAP	0.003	−0.002	0.008	*0.258*	0.003	−0.003	0.008	0.335
Aβ42/Aβ40	−0.003	−0.006	0.000	*0.080*	−0.004	−0.007	0.000	0.055
P‐tau181/AB42	0.003	−0.003	0.010	*0.343*	0.003	−0.004	0.010	0.358
PCA1	0.022	0.001	0.043	0.040	0.016	−0.007	0.039	0.181
PCA2	−0.032	−0.055	**−0.009**	**0.007**	−0.036	−0.062	−0.011	0.005

***Inc. dem vs. CU***	B	95% CI Lower Upper		*P*	B	95% CI Lower Upper		*P*
P‐tau181	0.0004	−0.008	0.009	*0.919*	−0.001	−0.009	0.007	0.832
NfL	0.003	−0.007	0.012	*0.569*	0.001	−0.010	0.011	0.903
GFAP	0.006	−0.002	0.014	*0.151*	0.005	−0.004	0.014	0.266
Aβ42/Aβ40	−0.003	−0.007	0.002	*0.263*	−0.002	−0.008	0.003	0.358
P‐tau181/AB42	−0.005	−0.016	0.006	*0.372*	−0.005	−0.018	0.008	0.416
PCA1	0.013	−0.024	0.050	0.498	0.002	−0.040	0.045	0.910
PCA2	−0.028	−0.063	0.007	0.119	−0.027	−0.068	0.014	0.200

***Inc. MCI vs. CU***	B	95% CI Lower Upper		*P*	B	95% CI Lower Upper		*P*
P‐tau181	0.005	−0.002	0.012	*0.159*	0.006	−0.001	0.014	0.087
NfL	0.002	−0.003	0.008	*0.439*	0.002	−0.004	0.008	0.537
GFAP	0.002	−0.004	0.008	*0.479*	0.002	−0.004	0.008	0.508
Aβ42/Aβ40	−0.003	−0.006	0.001	*0.109*	−0.004	−0.008	0.000	0.055
P‐tau181/AB42	0.006	−0.001	0.012	*0.114*	0.006	−0.001	0.013	0.105
PCA1	0.025	0.002	0.047	0.033	0.020	−0.005	0.044	0.115
PCA2	−0.033	−0.058	−0.008	0.010	−0.039	−0.067	−0.012	0.005

*Notes*: PCA1 and PCA2 were derived from principal component analysis (PCA) using log‐transformed values of Aβ42/Aβ40, p‐tau181/Aβ42, NfL, and GFAP, with PCA1 having positive loadings on p‐tau181/Aβ42, NfL, GFAP, and PCA2 having positive loading on Aβ42/Aβ40 and negative loading on p‐tau181/Aβ42.

Abbreviations: Aβ, amyloid beta; *APOE*, apolipoprotein E; BMI, body mass index; CI, confidence interval; CU, cognitively unimpaired; GFAP, glial fibrillary acidic protein; NfL, neurofilament light chain; p‐tau181, phosphorylated tau‐181.

^a^ Model 1: adjusted for age, sex, race/ethnicity, education.

^b^ Model 2: adjusted for age, sex, race/ethnicity, education, *APOE ε*4, Charlson Comorbidity Index.

^c^ B values in the table indicate the beta coefficient for the interaction between the disease status x time, with time being the duration (years) between the first blood visit to the follow‐up blood visits. Significant interactions indicate the rate of biomarker change over time in incident MCI and/or AD patients differ from the rate in cognitive unimpaired (CU) participants.

### 3.4 Sensitivity analysis

The GEE analyses results did not change when limiting analyses to the pre‐diagnosis visits only, that is, excluding the third visits of 94 individuals who had already developed MCI or dementia at the second visit, with a rapid decrease in PCA2 comparing incident MCI/AD (β = −0.034 [95% CI: −0.06, −0.008], *P* = 0.010) to CU adjusted for age, sex, ethnicity, education, *APOE* status, and comorbidities (Model 2).

When excluding CV% larger than 15%, the results are the same. When simultaneously adjusting for multiple comorbidities, the results remained similar (data not shown). We found sex, ethnic group, or *APOE* ɛ4 did not modify the association of biomarkers and disease outcome (*P* > 0.10 for all interaction terms; data not shown).

In a subset of the study population (*N* = 251), we found incident MCI/AD had a faster decline (b = −0.048 [95% CI: −0.080, −0.016], *P* = 0.003) in PCA2 compared to CU after adjusting for Model 2 covariates (age, sex, ethnic group, education, *APOE* status, and comorbidities) as well as creatinine and BMI.

## 4 DISCUSSION

In this community‐based cohort of CU adults, we found higher level of p‐tau181/Aβ42, and a biomarker pattern of higher level of p‐tau181/Aβ42 along with lower level of Aβ42/Aβ40 (i.e., PCA2), predicted the development of incident clinical AD. In addition, those who developed MCI/AD had a rapid decrease in Aβ42/Aβ40 along with an increase in p‐tau181/Aβ42, compared to participants who remained CU.

### 4.1 Predictive value of single measure of biomarkers

Our results provide important evidence that blood‐based AD biomarkers have clinical utility in predicting incident MCI and AD and in monitoring the cognitive trajectory among CU participants. We found p‐tau181 or p‐tau181/Aβ42 were the biomarkers most strongly associated with risk of cognitive impairment, consistent with previous studies.^5^, ^26^, ^39^ Although generally studies found biomarkers of the ATN and X (inflammation, etc.) framework are associated with increased risk of dementia, results for individual biomarkers other than p‐tau181 are not always consistent. In 300 participants of an Amsterdam study,^40^ both GFAP and Aβ42/Aβ40, but not NfL, were independently associated with incident dementia. In another study, GFAP showed the best performance, followed by NfL and p‐tau181, in predicting clinical AD risk.^41^ In the Rotterdam study,^42^ baseline NfL, Aβ42, and Aβ42/Aβ40 ratios, but not Aβ40 or t‐tau, were associated with risk of developing dementia. Overall, there is no consensus with regard to the relative importance of the biomarkers in predicting AD risk, but the significant findings are all in the expected direction, that is, increased biomarkers (or decreased Aβ42/Aβ40) are associated with increased risk of AD. Differences in sample size, age, sex, ethnic group, and comorbidities, factors that may influence the biomarker levels as found in the current study and others,^14^ may partially explain the inconsistent findings across studies. Recent studies evaluated the dynamic changes of the biomarkers along the AD continuum, and found GFAP might be an early AD biomarker, while p‐tau181 and NfL may subsequently predict AD at a later time.^43^, ^44^ Thus, inconsistent results from different studies might also be due to the different timing of blood sample collection.

### 4.2 Repeated measure of biomarkers

While biomarker levels in a one‐time measurement may help identify individuals at high risk of developing AD, monitoring the trajectory of biomarkers by repeated measurements might provide additional predictive value at an even earlier stage. An increase in the biomarker levels may indicate the beginning of the pathological process, and thus may provide a critical window for effective early prevention.^45^ We found all biomarkers, except for Aβ42/Aβ40, increased over time within individuals, consistent with the cross‐sectional findings of positive correlation between age and these biomarkers in the current study, as well as findings in previous studies that reported similar increase of biomarkers over time.^27^, ^39^, ^40^, ^42^, ^46^, ^47^, ^48^ However, we did not find a significant difference in the rate of change of the biomarkers comparing CU and those who developed cognitive impairments during follow‐up. Data are scarce in examining the rate of change of the biomarkers in relation to clinical disease status. In the Mayo Clinic Study of Aging (MCSA) study, the rate of increase in plasma NfL was not different between CU and MCI.^27^ In contrast, studies found mean plasma NfL levels, but not Aβ42^42^ or GFAP,^40^ increased faster in participants who developed dementia compared to participants who remained dementia‐free.^48^, ^49^ Additional evidence also supports that the increase in plasma NfL over time was associated with indicators of an active trajectory to MCI or AD, such as increasing level of amyloid PET^27^ and faster cognitive decline.^27^, ^48^ Although we did not find rate of NfL change varied between CU and MCI/AD, NfL did have a large contribution to the biomarker pattern PCA1, which increased at a marginally significantly faster speed in incident MCI than in CU.

Longitudinal changes of plasma p‐tau181 was found to be steeper in MCI than in CU,^39^ and was also associated with cognitive decline.^26^ In addition, increase in plasma p‐tau181 was related to the decrease in gray matter volume in certain brain areas^5^, ^50^ or amyloid deposition in the brain,^51^ which might stand as mediators leading to cognitive decline and dementia.^26^ In the current study, the biomarker pattern PCA2, with p‐tau181 and Aβ42/Aβ40 as the key components, showed different changes in MCI/AD compared to CU.

Overall, the biomarkers tended to have more rapid change among those developing MCI, but not those developing AD, compared to CU. One possible reason could be the biomarkers were already high at baseline, and thus may be closer to the “ceiling” and therefore slower change, in the AD patients.^44^

### 4.3 Combination of biomarkers

We found that most of the biomarkers were associated with the outcome in the expected direction, although after correction for multiple testing, some were not statistically significant. Studies found some AD biomarkers can provide non‐overlapping information on neuropathological changes,^52^ suggesting a holistic evaluation of the combined effect of the biomarkers may better capture the overall ATN and inflammation profile of an individual. Indeed, we found two patterns performed better than individual biomarkers in predicting incident dementia and monitoring development of MCI. Few previous studies combined multiple biomarkers.^42^, ^53^, ^54^ Similar to the pattern PCA2 in our study, the Rotterdam study^42^ found combining the lowest quartile group of Aβ42 with the highest of NfL resulted in a stronger association with dementia, compared to the highest quartile group of Aβ42 and lowest of NfL. Combining p‐tau217 and the Aβ42/40 ratio showed the highest accuracy for predicting the presence of AD pathological changes, outperforming single biomarkers.^55^ A recent study found a two‐step workflow, using plasma p‐tau217 to screen for Aβ positivity in step 1 and CSF Aβ42/Aβ40 in step 2, was a highly accurate and cost‐effective strategy to detect AD in memory clinic settings.^56^ Moreover, combining Aβ42/Aβ40 and plasma GFAP, with age and *APOE* status, provided the optimal panel identifying a positive amyloid status.^53^ Similarly, a stronger association with incident dementia was found for joint NfL and GFAP compared to either of the two individual biomarkers.^54^ Overall, while there is no consensus of the best combination of the biomarkers, these studies point to an increased value of examining the biomarkers simultaneously, compared to individual ones, in dementia research. As AD is known to be a complex multi‐factorial neurodegenerative disorder,^57^ combining biomarkers measuring different pathways may indeed be necessary in future studies.

### 4.4 Limitations and advantages

While many studies focused on dichotomizing biomarker values based on cutoff points, we used the full range of each biomarker to assess risk of developing clinical MCI or AD. We show that there is a linear relationship between increased p‐tau181 or p‐tau181/Aβ42 ratio, which would not be confirmed if a dichotomous cut point had been used. Thus, there is a clear disadvantage in using derived cut points to assess risk of disease because there is a loss of potentially valuable information. We did not measure other p‐tau isoforms (p‐tau217, p‐tau231, p‐tau205, p‐tau212) because they were not commercially available when the study began. They may have shown significant associations with cognitive decline in non‐demented subjects.^58^, ^59^ However, the clinical and analytical performances of different species of p‐tau assays have been shown to be largely comparable and their values correlated strongly with each other.^60^ While we adjusted for multiple key factors including age, sex, ethnic group, and *APOE*, we did not have other putative confounders, such as creatinine and BMI, in the entire study population. However, to be consistent with the literature,^61^ we adjusted for these variables in the subset but with similar results. Although this study is relatively large and has repeated measures of biomarkers, only a small number of White non‐Hispanic and Black individuals developed AD; thus, our statistical power to detect significant results in those groups was limited.

Our study has many strengths. Our study population was from a community‐based, multi‐ethnic cohort, and thus may have good representation of general population. We measured both biomarkers and outcome longitudinally, with three measures of biomarkers in most participants and the follow‐up time up to 23 years. We adjusted for potential confounders. In addition to examining individual biomarkers, we derived two biomarker patterns, which were quite robust across different visits and showed stronger association with outcomes than individual biomarkers.

While many studies use autopsy or PET imaging to establish optimal thresholds or cut points for the diagnosis of AD, there are still no universal or established cut points for the use of these AD biomarkers as diagnostics. However, in this investigation, we found that AD biomarkers collected longitudinally may be clinically useful as adjuncts to neurological and cognitive evaluations. Previous cross‐sectional studies have concluded that these AD biomarkers provide a physiological basis for the diagnosis of AD consistent with the ATN recommendations. Here we did not determine, nor did we include, thresholds or cut points; rather, we used the AD biomarkers to determine whether they are consistent with the clinical diagnosis. Advances in therapeutic strategies for AD need to include risk prediction. The AD biomarkers used here represent a reasonable approach to risk prediction.

## CONFLICT OF INTEREST STATEMENT

The authors declare no conflicts of interest. Author disclosures are available in the supporting information.

## CONSENT STATEMENT

All individuals participating in the study provided written informed consent.

## Supporting information

Supporting Information

Supporting Information
