# Supplementary material for: Risk of Alzheimer's disease is associated with longitudinal changes in plasma biomarkers in the multi‐ethnic Washington Heights–Hamilton Heights–Inwood Columbia Aging Project (WHICAP) cohort
Source: Alzheimers Dement. 2024 Jan 6;20(3):1988–99. doi: 10.1002/alz.13652 (PMC10984426; doi:10.1002/alz.13652)
Supplement: Supplementary file 1 — Supporting Information [file ALZ-20-1988-s002.docx]

**Supplementary Table 1. Biomarker patterns derived from principal component analyses.**

|  | visit 1 | | visit 2 | | visit 3 | |
| --- | --- | --- | --- | --- | --- | --- |
|  | PCA1 | PCA2 | PCA1 | PCA2 | PCA1 | PCA2 |
| Aβ42/40 ratio |  | 0.957 |  | 0.872 |  | 0.868 |
| P-tau181/Aβ42 ratio | 0.482 | -0.303 | 0.367 | -0.647 | 0.356 | -0.683 |
| NfL | 0.854 |  | 0.853 |  | 0.835 |  |
| GFAP | 0.808 |  | 0.863 |  | 0.867 |  |

The patterns were derived from three visit-specific principal component analyses, each having the first two patterns (PCA1 and PCA2) retained. Loadings less than 0.3 were omitted in the table.
